# Alterations of Spinal Epidural Stimulation-Enabled Stepping by Descending Intentional Motor Commands and Proprioceptive Inputs in Humans With Spinal Cord Injury

**DOI:** 10.3389/fnsys.2020.590231

**Published:** 2021-01-28

**Authors:** Megan L. Gill, Margaux B. Linde, Rena F. Hale, Cesar Lopez, Kalli J. Fautsch, Jonathan S. Calvert, Daniel D. Veith, Lisa A. Beck, Kristin L. Garlanger, Dimitry G. Sayenko, Igor A. Lavrov, Andrew R. Thoreson, Peter J. Grahn, Kristin D. Zhao

**Affiliations:** ^1^Assistive and Restorative Technology Laboratory, Department of Physical Medicine and Rehabilitation, Rehabilitation Medicine Research Center, Mayo Clinic, Rochester, MN, United States; ^2^Mayo Clinic Graduate School of Biomedical Sciences, Mayo Clinic, Rochester, MN, United States; ^3^Department of Neurosurgery, Center for Neuroregeneration, Houston Methodist Hospital, Houston, TX, United States; ^4^Department of Neurology, Mayo Clinic, Rochester, MN, United States; ^5^Institute of Fundamental Medicine and Biology, Kazan Federal University, Kazan, Russia; ^6^Department of Neurosurgery, Mayo Clinic, Rochester, MN, United States; ^7^Office for Education Diversity, Equity and Inclusion, Mayo Clinic, Rochester, MN, United States; ^8^Department of Physiology and Biomedical Engineering, Mayo Clinic, Rochester, MN, United States

**Keywords:** paralysis, spinal cord injury, task-specific training, multi- modal rehabilitation, body weight supported stepping, spinal neuromodulation, epidural spinal stimulation

## Abstract

**Background:** Regaining control of movement following a spinal cord injury (SCI) requires utilization and/or functional reorganization of residual descending, and likely ascending, supraspinal sensorimotor pathways, which may be facilitated via task-specific training through body weight supported treadmill (BWST) training. Recently, epidural electrical stimulation (ES) combined with task-specific training demonstrated independence of standing and stepping functions in individuals with clinically complete SCI. The restoration of these functions may be dependent upon variables such as manipulation of proprioceptive input, ES parameter adjustments, and participant intent during step training. However, the impact of each variable on the degree of independence achieved during BWST stepping remains unknown.

**Objective:** To describe the effects of descending intentional commands and proprioceptive inputs, specifically body weight support (BWS), on lower extremity motor activity and vertical ground reaction forces (vGRF) during ES-enabled BWST stepping in humans with chronic sensorimotor complete SCI. Furthermore, we describe perceived changes in the level of assistance provided by clinicians when intent and BWS are modified.

**Methods:** Two individuals with chronic, mid thoracic, clinically complete SCI, enrolled in an IRB and FDA (IDE G150167) approved clinical trial. A 16-contact electrode array was implanted in the epidural space between the T11-L1 vertebral regions. Lower extremity motor output and vertical ground reaction forces were obtained during clinician-assisted ES-enabled treadmill stepping with BWS. Consecutive steps were achieved during various experimentally-controlled conditions, including intentional participation and varied BWS (60% and 20%) while ES parameters remain unchanged.

**Results:** During ES-enabled BWST stepping, the knee extensors exhibited an increase in motor activation during trials in which stepping was passive compared to active or during trials in which 60% BWS was provided compared to 20% BWS. As a result of this increased motor activation, perceived clinician assistance increased during the transition from stance to swing. Intentional participation and 20% BWS resulted in timely and purposeful activation of the lower extremities muscles, which improved independence and decreased clinician assistance.

**Conclusion:** Maximizing participant intention and optimizing proprioceptive inputs through BWS during ES-enabled BWST stepping may facilitate greater independence during BWST stepping for individuals with clinically complete SCI.

**Clinical Trial Registration:** ClinicalTrials.gov identifier: NCT02592668.

## Introduction

A spinal cord injury (SCI) causes disruption of communication between spinal circuitries and supraspinal centers often resulting in permanent motor and sensory deficits. Advanced rehabilitation approaches, such as task-specific training, focus on re-engaging spinal circuitries below the level of injury to gain recovery of lost motor and sensory functions with a goal of increasing independence for individuals with spared motor or sensory function (Behrman et al., 2017). Regaining control of goal directed intentional movement following SCI requires utilization and/or functional reorganization of residual descending, and likely ascending, supraspinal sensorimotor pathways (Winstein et al., 1994; Winchester et al., 2005; Cai et al., 2006; Field-Fote and Roach, 2011; Petersen et al., 2012; Barthélemy et al., 2015; Huie et al., 2017). More specifically, body weight supported treadmill (BWST) training is used by clinicians to facilitate spinal circuitry influencing afferent proprioceptive input in a task-specific manner, which has been demonstrated to improve locomotor functions in individuals with motor incomplete SCI (Thomas et al., 2005). However, BWST training has not been shown to sufficiently facilitate functional residual connections in individuals with motor complete SCI (Forrest et al., 2008; Scivoletto et al., 2014).

During BWST training, spinal circuitries below the injury site are capable of interpreting afferent proprioceptive inputs in order to coordinate downstream motor outputs during activities such as standing and stepping (Harkema et al., 1997; Dietz et al., 2002; Beres-Jones and Harkema, 2004; Edgerton et al., 2008). Activation of proprioceptive inputs can be achieved during BWST training through lower extremity loading, clinician-assisted joint manipulation and tactile facilitation (Harkema et al., 2011a). Specifically, lower extremity loading has been shown to increase extensor muscle activity in individuals with SCI during stepping, even those with motor complete SCI, despite no return of clinically detectable function (Dietz et al., 1995; Dobkin et al., 1995; Harkema et al., 1997; Wirz et al., 2001; Apte et al., 2018).

Traditionally in the field of rehabilitation, patient progress is measured through the level of perceived independence determined by the amount of physical assistance needed to complete activities of daily living such as walking. Quantitative measures to describe performance during a dynamic task such as BWST stepping in the motor complete SCI population is challenging. The level of clinician assistance for joint manipulation and tactile facilitation are constantly changing, determined by the success of generating flexion or extension movements necessary for each phase of gait. Galvez et al. (2011) quantified trainer/clinician variability of manual skills during BWST step training while identifying key phases of gait with the greatest amount of variability.

Over the last decade, investigations of task-specific training combined with epidural electrical stimulation (ES) applied to the dorsal surface of the spinal cord, below the level of SCI, have demonstrated the restoration of standing and stepping functions in individuals diagnosed as motor complete SCI (Rejc et al., 2015, 2017; Grahn et al., 2017; Angeli et al., 2018; Gill et al., 2018). Functional improvements enabled by ES and task-specific training are thought to be achieved by facilitating activity across spinal circuitries in order to re-establish states of excitability that enable robust, coordinated motor outputs necessary to perform standing and stepping tasks (Dimitrijevic et al., 1998; Minassian et al., 2004; Danner et al., 2015). Our team previously reported the use of multi-modal rehabilitation, a combined approach of task-specific training with continuous ES, in an individual with a sensorimotor complete SCI, which resulted in the ability to step over ground with the aid of a walker and minimal assistance at the hips for balance and no assistance at the knees (Gill et al., 2018). In the presence of ES, improvements of motor functions were observed over 12 months of BWST training. These improvements were dependent upon several variables such as optimization of proprioceptive input during training, ES parameters and the degree to which participants attempted to intentionally control motor activity during each step cycle. However, the impact of each variable on the degree of independence achieved during BWST stepping remains unknown.

Herein, we describe lower extremity motor activity and vertical ground reaction forces (vGRF) during various experimentally-controlled conditions of ES-enabled BWST stepping in two individuals with chronic, complete loss of function below the level of SCI. Furthermore, we describe qualitative features of perceived changes in the level of assistance provided by clinicians during ES-enabled BWST stepping.

## Methods

### Participants

Two participants diagnosed with an American Spinal Injury Association (ASIA) Impairment Scale Grade A (AIS-A) sensorimotor complete SCI (Burns et al., 2012) were enrolled in this clinical trial. Participant 1, 26 years of age, sustained traumatic T6 SCI 3 years prior to enrollment. Participant 2, age 37 years of age, sustained traumatic T3 SCI 6 years prior to enrollment. Both participants presented with absent lower extremity motor evoked potentials and scalp somatosensory evoked potentials. Both participants demonstrated evidence of spared connections of lower extremity non-specific electromyography (EMG) during Jendrassik maneuver described in the literature as a discomplete SCI profile (Dimitrijevic, 1988; Sherwood et al., 1992). Participants provided written, informed consent to all procedures which were performed under the approval of the Mayo Clinic Institutional Review Board with a US Food and Drug Administration Investigational Device Exemption (IDE G150167).

### Clinical Trial Protocol

Following 6 months of locomotor training (Harkema et al., 2011b) both participants were implanted with the Medtronic® Specify 5-6-5 spinal epidural electrode array (Medtronic, Fridley, MN) which was internally connected to the RestoreUltra SureScan MRI Neurostimulator (Model 97712, Medtronic, Fridley, MN). After recovering from surgery, ES-enabled task-specific training was performed over a period of ~12 months (Gill et al., 2018). Following 12 months of ES-enabled task-specific training, participants returned for a data collection session aimed at comparing BWST stepping conditions (e.g., participant intent and varied BWS) with consistent ES parameters.

### ES Parameter Selection

During initial sessions of ES-enabled task-specific training, stimulation parameters were adjusted incrementally while recording lower extremity EMG synchronized to delivery of each ES pulse in order to examine lower extremity muscle recruitment curves (Sayenko et al., 2014; Grahn et al., 2017; Calvert et al., 2019b). ES parameter usage at supra-motor threshold levels, defined as voltages that evoked observable activity in skin surface EMG recordings that were robust enough to generate movement of the lower extremities (Dimitrijevic et al., 1998), were utilized during the initial sessions of BWST training (Grahn et al., 2017; Calvert et al., 2019a). Subsequent refinement of these parameters occurred during each session of BWST training with an overarching goal of maximizing independent, volitional control over lower extremity movements comprised of stepping characteristics (e.g., initiation and/or termination of swing phase during contralateral weight bearing stance phase) (Gill et al., 2018). Refinement across BWST sessions resulted in a narrowed range of ES parameters with respect to voltage amplitude (2.0–4.1 V), pulse frequency (20–30 Hz), and pulse width (200–450 μs) applied continuously. During stepping experiments reported herein, ES parameters identified at the beginning of the data collection session were not adjusted across conditions.

### Experimental Conditions

Multi-modal rehabilitation during the initial 12 months focused on standing and stepping utilizing a BWST system, along with a computer-controlled motorized treadmill, as well as a team of clinicians with expertise in assisting joint manipulation and tactile facilitation consistent with locomotor training principles (Behrman and Harkema, 2000; Beres-Jones and Harkema, 2004; Dolbow et al., 2015; Behrman et al., 2017). Participant-specific ES parameters and treadmill speeds, which were considered optimal for achieving the greatest independence during BWST stepping, remained unchanged during the data collection session. Participants were allowed to use parallel bars during BWST stepping to facilitate trunk stability and manipulation of weight shifts in an effort to maximize independence. However, participants were instructed to refrain from using their hands on parallel bars for weight-bearing. Changes in BWS were monitored using the treadmill software (Power Neuro Recovery, Louisville, KY, USA). Additionally, during passive conditions participants were asked to simply rest their arms on the bars and not engage in weight shifting. Stepping assistance was provided as needed at the hips, knees, and ankles. The order between intent and BWS conditions was standardized for both participants during each testing condition in the following manner: (1) 60% BWS, (2) 20% BWS, then (3) active stepping, and (4) passive stepping.

#### Intent Conditions (Active and Passive Stepping)

Here, intent describes descending commands and is defined as the intentional participation utilized during active stepping conditions. Methods to engage intentional participation included visual feedback while using mirrors and verbal feedback both between clinicians and to the participants for optimal kinematics during stepping bouts. Participants were instructed to fully concentrate on achieving both stance and swing phases of each gait cycle. Passive stepping occurred when the participants were instructed to have no intent while clinicians facilitated stepping movements as needed. Furthermore, participants were instructed to completely relax and allow the clinicians to assist with the stepping task. To compare motor outputs between active and passive stepping conditions, the participants were asked to complete 10 consecutive steps bilaterally for each condition. ES parameters, BWS (20%), and treadmill speed (0.5 mph) remained unchanged during testing bouts. Clinician assistance was provided as needed to successfully complete each step.

#### BWS Conditions (60% and 20% Unloading)

BWS describes afferent proprioceptive input based on the percentage of the participant's body weight offloaded during BWST stepping. To compare motor output differences between two considerably different loading environments, 60% and 20% BWS levels were utilized. The participants were instructed to fully concentrate on achieving 10 consecutive steps bilaterally for each BWS condition, and actively focus on achieving appropriate stance and swing phases. ES parameters, participant intent (active stepping), and speed (0.5 mph) remained unchanged during testing bouts. Clinician assistance was provided as needed to successfully complete each step.

### Data Collection

Stepping data was collected at a single time point following 12 months of ES-enabled task-specific training. Motor outputs for the 10 consecutive bilateral steps in each condition were collected using skin surface EMG recorded bilaterally on the rectus femoris (RF), vastus lateralis (VL), medial hamstring (MH), medial gastrocnemius (MG), soleus (SOL), and tibialis anterior (TA) muscles at a sampling rate of 4 kHz (PowerLab, AD Instruments, Inc., Colorado, USA). vGRF was recorded using shoe insole pressure sensors at a sampling rate of 50 Hz (FSCAN, Tekscan, Inc., South Boston, MA, USA). Data were synchronized with real time video capture using Labchart electrophysiological software.

### Clinician Perceived Level of Assistance

**Figure 1:**
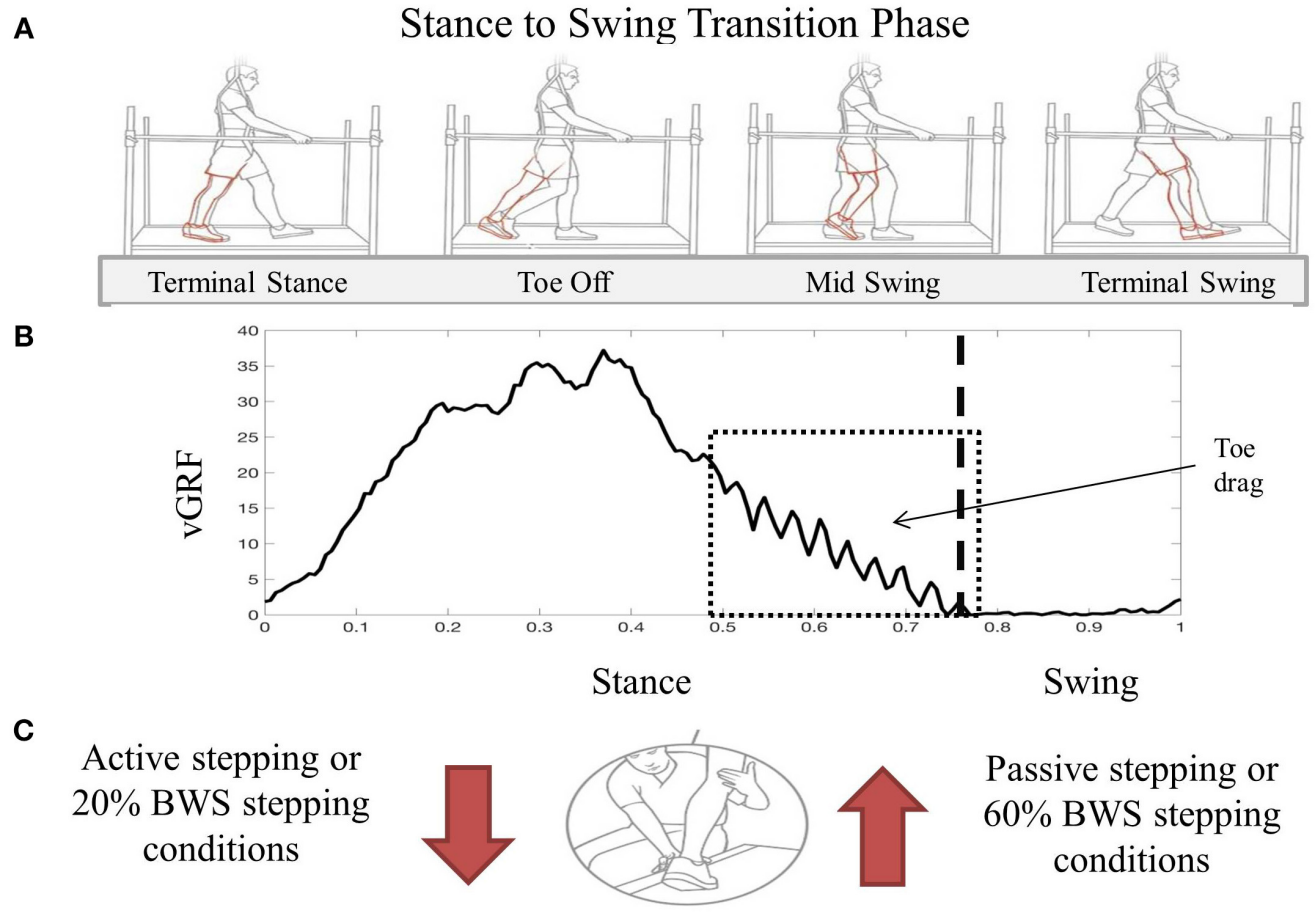
Visual description of transition from stance to swing during BWST stepping **(A)**. Red limb indicates inadequate movement of the limb during swing phase demonstrating increased knee extension during toe off and toe drag during mid swing. An exemplary vGRF trace during one step cycle demonstrating increased difficulty transitioning from stance to swing with oscillatory force pattern representing toe drag **(B)**. Box represents the time during the gait cycle where assistance level increased as perceived by the clinicians assistance level increased as perceived by the clinicians. During the transition from stance to swing, clinician assistance facilitation technique increased during passive and 60% BWS stepping conditions compared to active and 20% BWS stepping **(C)**. Image used in Panel A was created by Mayo Clinic's Medical Illustration Department.

During stepping, clinician assistance varied based on success in achieving each phase of the gait cycle. Assistance levels were reported by the clinicians and available reviewed through video recordings. Using locomotor training principles, clinician-assisted joint manipulation and tactile facilitation were used to activate appropriate muscles during specific gait phases for knee control, flexion vs. extension movements, and ankle control for toe clearance and foot placement (Harkema et al., 2011a). Knee control during stance requires facilitation at the anterior tibial crest with dual purpose of knee extension force and facilitation of the patellar tendon with the goal of engaging the knee extensor muscles. Knee control during swing phase responds to a quick stretch of the hamstring tendon to facilitate knee flexion. During stance phase, ankle control assistance is necessary for foot placement and to stabilize against rotation during loading. Clinicians performing the data collection were consistent but not blinded to the condition. Subjective reporting of either an increase or decrease in assist level, as well as identification of the phase of gait perceived to change, was not based on any scale and was verbally reported after data collection. These methods have not been validated and rely on the clinician's vast experience following 12 months of multi-modal rehabilitation with each participant. Between conditions, the level of clinician assistance was most variable during the transition from stance to swing phase (Figure 1).

### Data Analysis

Data was processed using a custom MATLAB algorithm (MATLAB, The MathWorks Inc., Natick, MA, USA). A total of 10 steps were collected from each participant in each condition. Five steps from participant 2 were excluded due to inconsistent use of the upper extremities. Data were averaged by participant, right or left limb, and stepping condition. Only the RF and/or the VL muscles were selected to be analyzed due to role of each during the stance phase of gait. EMG activation was also averaged by muscle. EMG data were full wave rectified and filtered with a second order zero-phase lag band-pass filter for frequencies between 59 and 61 Hz to remove any electrical noise. Subsequently, the linear envelope was created by low-pass filtering at 3 Hz using a zero-phase lag second order Butterworth filter (Olney and Winter, 1985; Arendt-nielsen, 1994; Heintz and Gutierrez-farewik, 2007; Danner et al., 2015; Lerner et al., 2017). The root mean square (RMS) was calculated from mean EMG responses of individual muscles in each condition for the stance and swing phases separately. A statistical analysis was performed using JMP (Cary, NC, USA) to determine if differences between RMS values were significant between conditions in each participant. Paired *t*-tests were performed on the VL and RF RMS values between passive and active stepping and 60% and 20% BWS stepping within each participant. Alpha was set to 0.05. Percent of the gait cycle was determined using a modified threshold equation on the vGRF signal (FSCAN, Tekscan) and verified though 2D video analysis. EMG and vGRF signals for each step were normalized from 0 to 100% of the gait cycle. The time of transition from stance to swing was calculated based on proprietary algorithms within the Tekscan software. A stance to swing transition phase was calculated as 60–90% of the gait cycle. Right and left sides were processed identically.

## Results

### Influence of Intent on Stepping

#### Clinician Perceived Level of Assistance

The clinician perceived level of assistance for both participants was less during the transition phase from stance to swing during active stepping when compared to passive stepping. During active stepping, the transition from stance to swing was timely and purposeful, reducing the level of clinician assistance. When each participant was passively stepping, the stance to swing transition resulted in excessive lower extremity extension which requiring increased clinician assistance to facilitate flexion in order to minimize toe drag (Figure 1). Due to the decrease in trainer assistance, active stepping resulted in more independence than passive stepping.

#### vGRF

**Figure 2:**
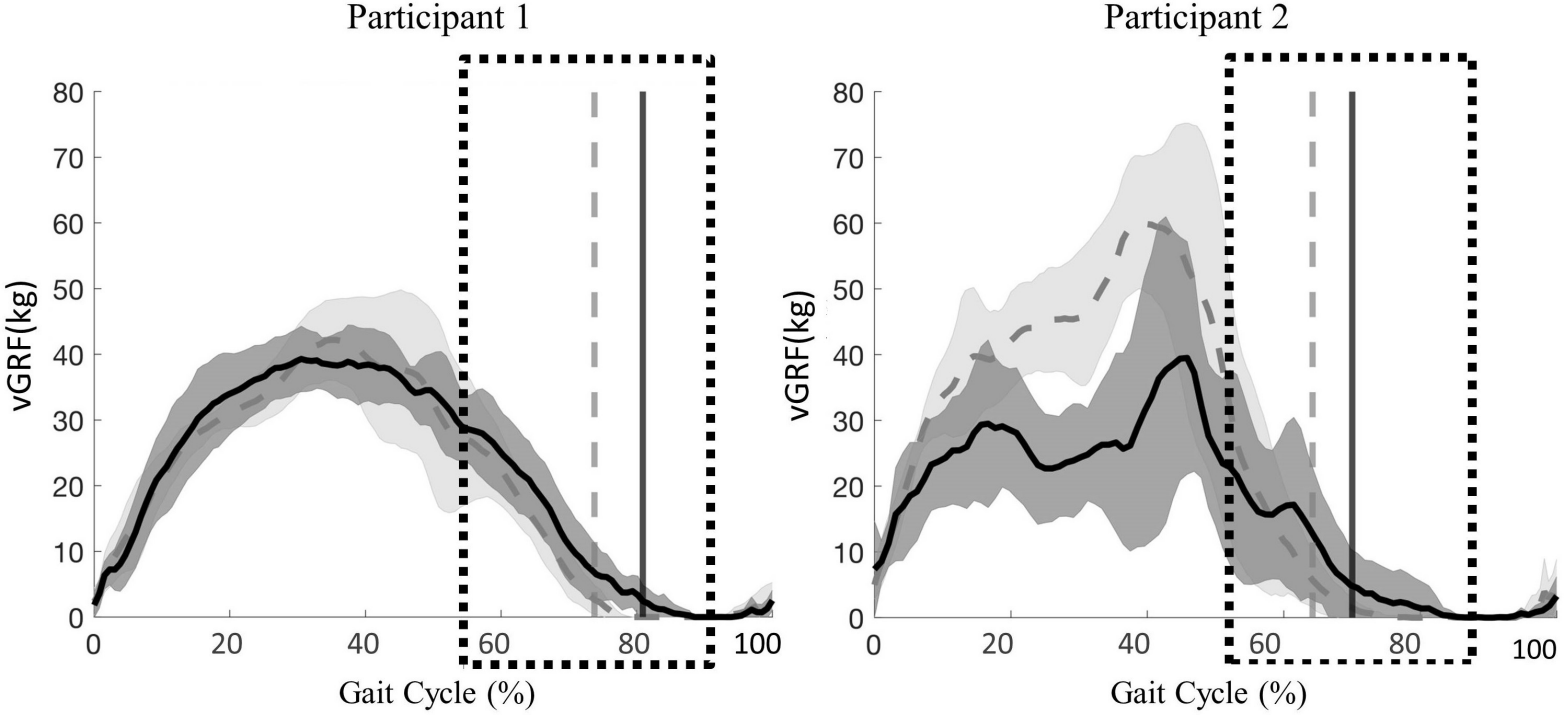
Participant 1(right limb) and participant 2 (left limb), mean ± *SD* vGRF during passive (solid line) and active (dashed line) stepping across the gait cycle. Vertical lines represent transition from stance to swing for each respective condition. Boxes represent time during the gait cycle where assistance level increased as perceive by the clinicians.

Peak vGRF timing over the entire gait cycle and vGRF magnitude during the transition phase of stance to swing were comparable between active and passive stepping conditions. For participant 1, the transition phase occurred on average 6% (right and left limb) later in the gait cycle in passive stepping when compared to active stepping (Figure 2). For participant 2, the transition phase occurred on average 2% and 6% (right and left limb) later in the gait cycle in passive stepping when compared to active stepping (Figure 2). Both participants exhibited toe drag in passive stepping as indicated by the oscillating mean curve from 70% to 100% of the gait cycle. Passive stepping toe drag was also verified with 2D video analysis. Active stepping did not result in toe drag.

#### Lower Extremity EMG

**Figure 3:**
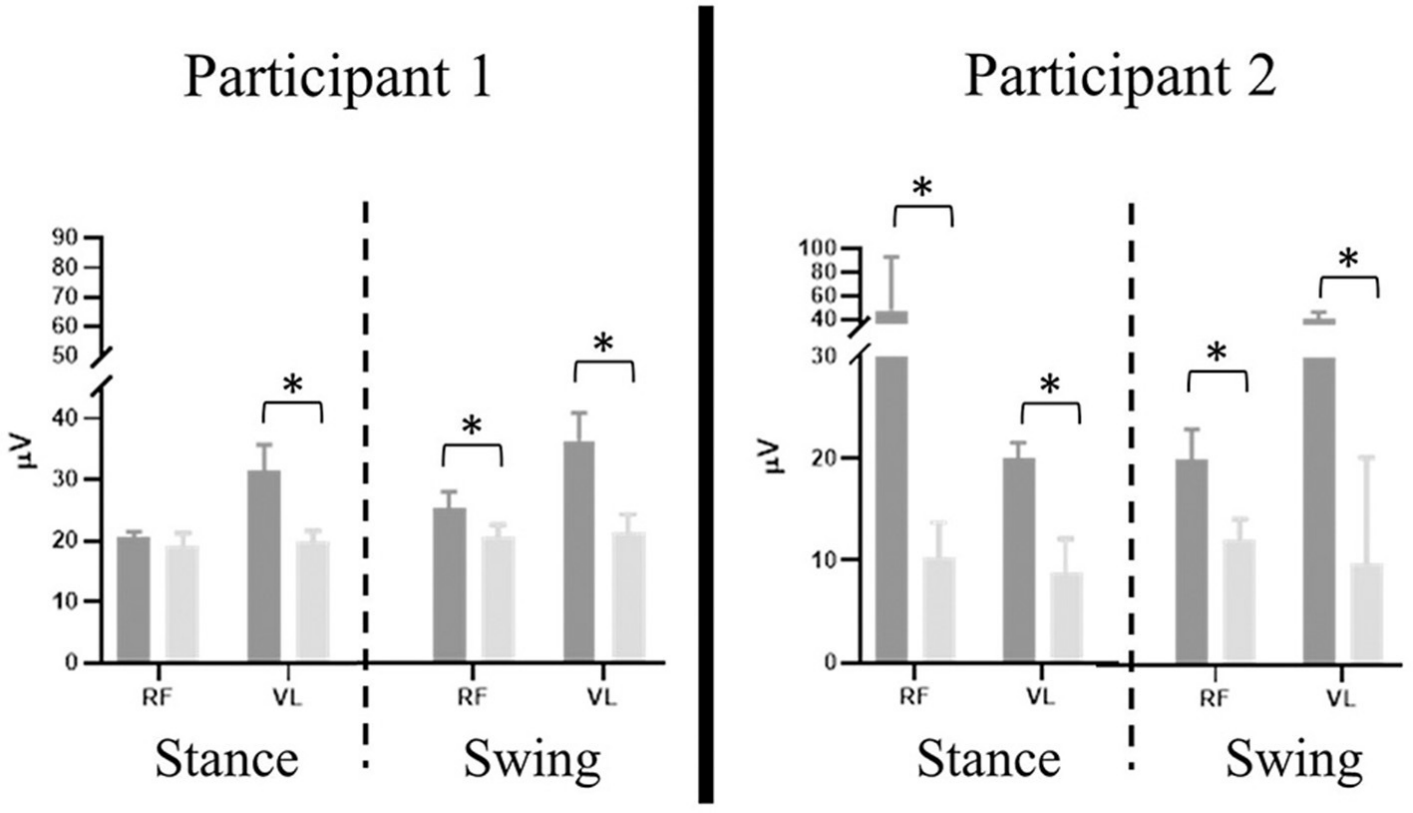
Participant 1 (right limb) and participant 2 (left limb), mean ± *SD* RMS values of rectus femoris (RF) and vastus lateralis (VL) results separated by stance and swing phase (dashed line) during passive (dark gray) and active (light gray) stepping. Error bars represent one standard deviation. Asterisk denotes *P* < 0.01.

**Figure 4:**
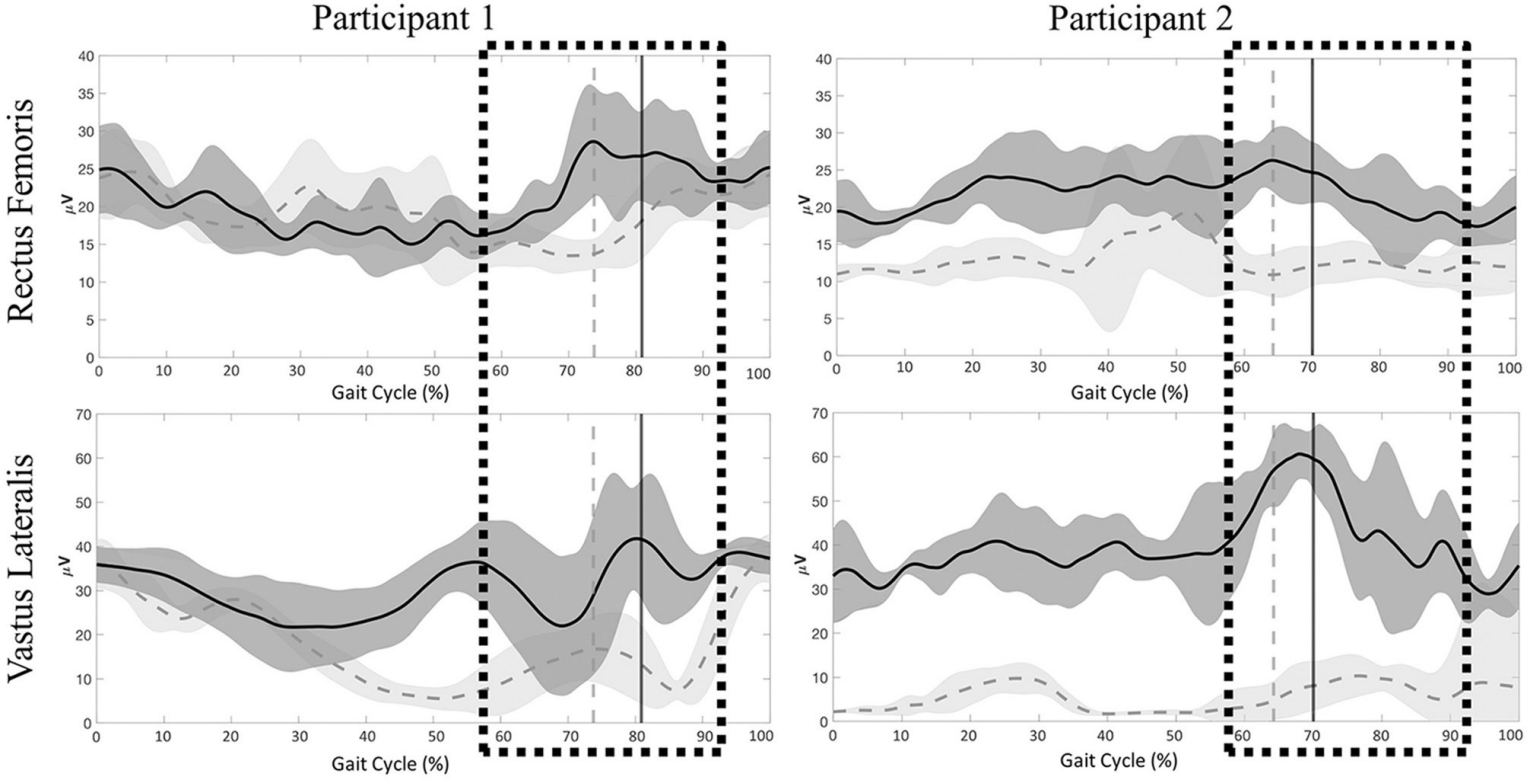
Participant 1 (right limb) and participant 2 (left limb), mean ± *SD* electromyography activation of the rectus femoris (RF) and vastus lateralis (VL) for Participant 1 and Participant 2 during passive (dark gray) and active (light gray) stepping conditions. Vertical lines represent transition from stance to swing for each respective condition. Boxes represent time during the gait cycle were clinicians perceived an increase in the level of assistance.

Significant differences were present in the knee extensor RMS values during passive stepping when compared to active stepping during both stance and swing phase (*P* < 0.01) (Figure 3). RF RMS values during passive stepping were higher than in active stepping (participant 1: 1.81 μV higher in stance and 4.39 μV higher in swing; participant 2: 15.48 μV higher in stance and 13.6 μV higher in swing). VL RMS values during passive stepping were higher than in active stepping (participant 1: 10.8 μV higher in stance and 15.9 μV higher in swing; participant 2: 33.1 μV higher in stance and 36.7 μV higher in swing). Participant 1 demonstrated constant RF activation during the full gait cycle in both active and passive stepping (Figure 4). During the transition from stance to swing (60–90% of the gait cycle) passive stepping resulted in a larger increase in RF activation in comparison to active stepping. Similar activation patterns emerged in the VL; decreases in activation were larger during active stepping compared to passive stepping. The RF and VL for participant 2 also remained constant during stance to swing transition much greater activation observed for passive stepping compared to active stepping. Overall, the RF and VL for both participants were greater during the transition from stance to swing during passive stepping compared to active. Changes between 20 and 60% BWS in the medial hamstring and distal muscles were minimal. Data can be viewed in the Supplementary Material.

### Effect of Altering Body Weight Support During Stepping

#### Clinician Perceived Level of Assistance

Clinician perceived level of assistance for both participants during the transition from stance to swing phase was less during 20% BWS stepping than during 60% BWS stepping. For both participants, the transition from stance to swing was timely and purposeful, demonstrating appropriate lower extremity movement from extension during stance to flexion during swing (Figure 1). The clinicians reported less assistance required for stepping bouts during 20% BWS.

#### vGRF

**Figure 5:**
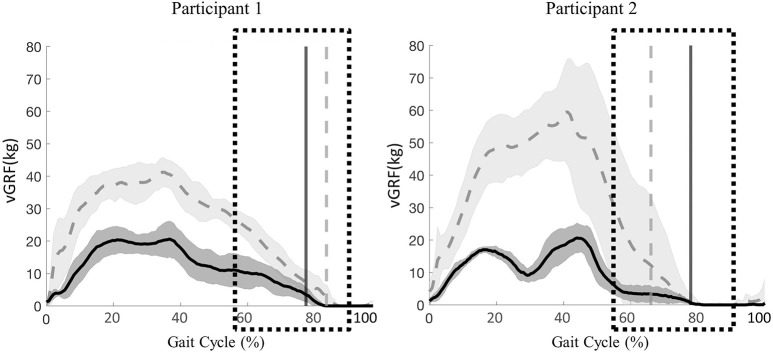
Participant 1 (right limb) and participant 2 (left limb), mean ± *SD* vGRF during 60% (solid line) and 20% (dashed line) BWS stepping across the gait cycle. Vertical lines represent transition from stance to swing for each respective condition. Boxes represent time during the gait cycle where clinicians perceived an increase in the level of assistance.

The peak vGRF during 20% BWS stepping was at least twice the magnitude of that for 60% BWS stepping (Figure 5). Participant 1 exhibited a later transition from stance to swing in 20% BWS stepping when compared to 60% BWS stepping (2% of the gait cycle). Whereas, participant 2 demonstrated a transition phase 11% later in the gait cycle in 60% BWS stepping when compared to 20%. Toe drag was not visibly different in 60% and 20% BWS stepping conditions.

#### Lower Extremity EMG

**Figure 6:**
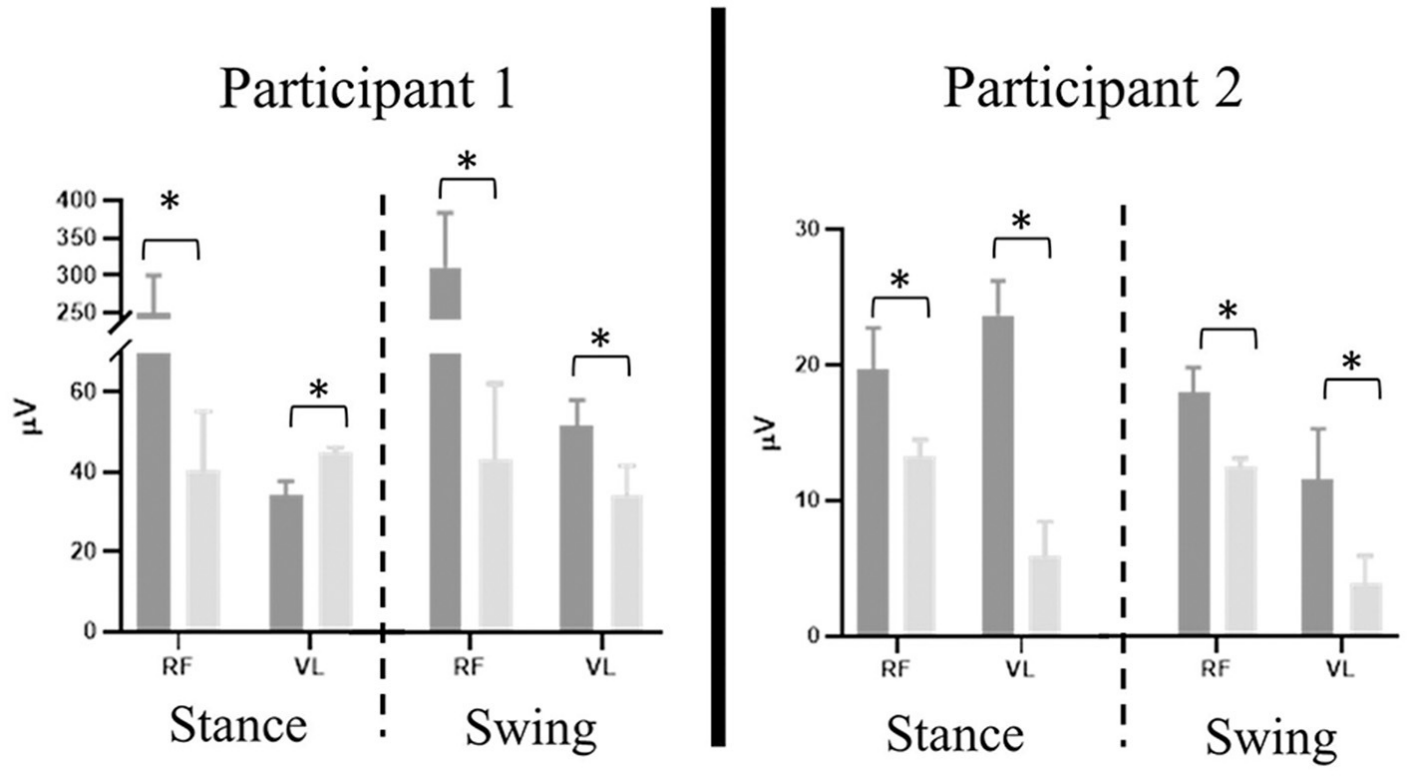
Participant 1 (right limb) and participant 2 (left limb), mean ± *SD* RMS values of rectus femoris (RF) and vastus lateralis (VL) results separated by stance and swing phase (dashed line) during 60% (dark gray) and 20% (light gray) BWS stepping. Error bars represent one standard deviation. Asterisk denotes *P* < 0.01.

**Figure 7:**
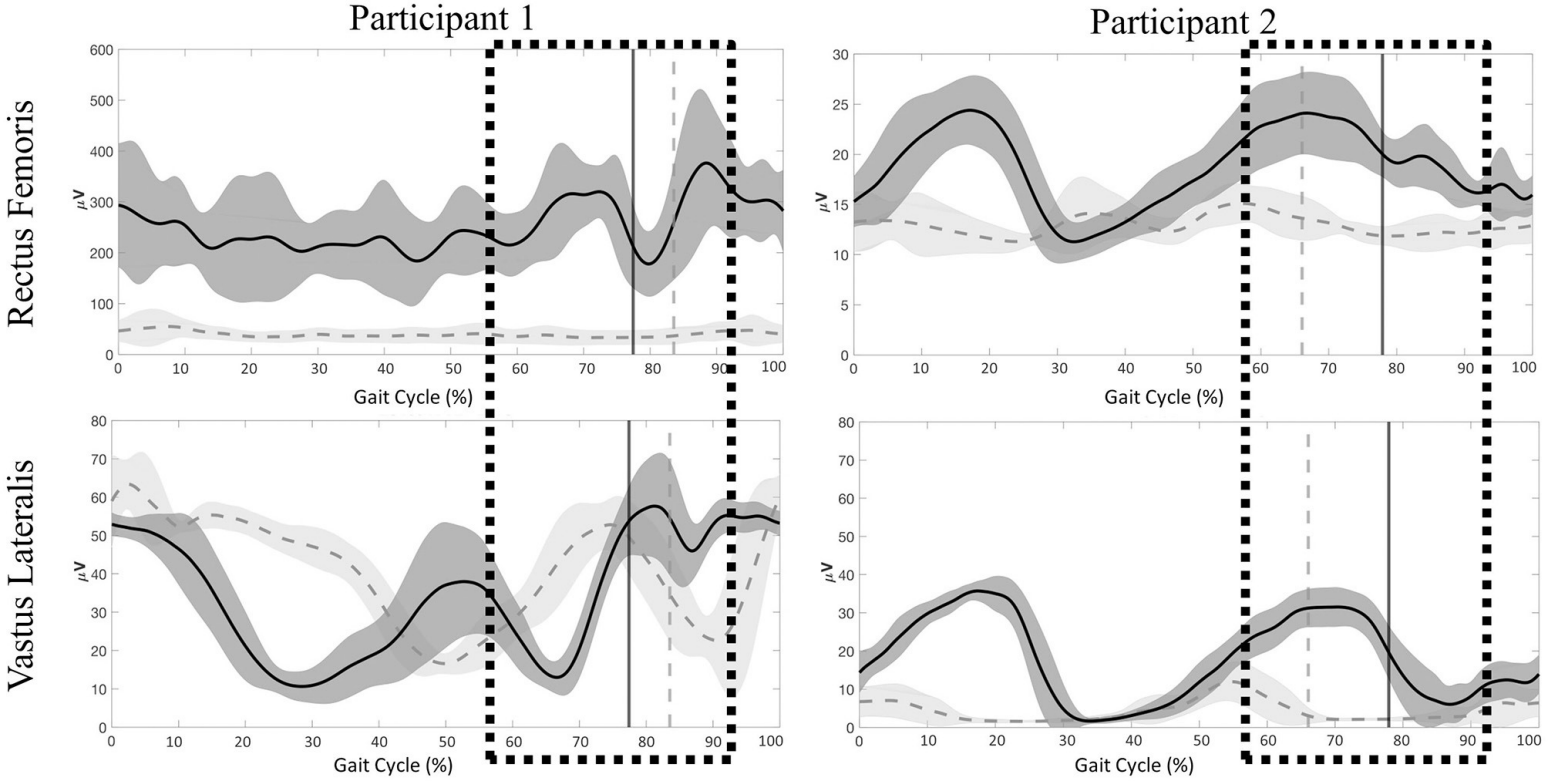
Participant 1 (right limb) and participant (left limb), mean ± *SD* electromyography activation of the rectus femoris (RF) and vastus lateralis (VL) during 60% (dark gray) and 20% (light gray) BWS stepping conditions. Vertical lines represent transition from stance to swing for each respective condition. Boxes represent time during the gait cycle were clinicians perceived an increase in the level of assistance.

Stepping at 20% BWS, when compared to 60% BWS, resulted in significantly lower RMS values during both stance and swing phases (*P* < 0.01) with the exception of the VL for participant 1 (Figure 6). The RF RMS values were significantly higher in 60% BWS stepping when compared to 20% BWS stepping (participant 1: 203.8 μV higher in stance and 261.9 μV higher in swing; P2: 6.36 μV higher in stance and 5.52 μV higher in swing; *P* < 0.01). During stance phase, participant 1 VL RMS was 8.7 μV less in 60% BWS stepping when compared to 20% BWS stepping. However, in swing phase VL RMS was 15.17 μV greater in 60% BWS stepping when compared to 20% BWS stepping. During both stance and swing phase, participant 2 demonstrated significantly higher VL RMS in 60% BWS stepping when compared to 20% (17.3 μV higher in stance and 6.52 μV higher in swing). Participant 1 RF demonstrated a sharp decreased in activation as toe off occurred during 60% BWS stepping whereas during 20% BWS stepping RF activation remained constant. Overall activation during 60% BWS stepping was much higher, especially during the transition from stance to swing phase compared to 20% BWS stepping (Figure 7). In participant 1 a large increase in VL activation was observed just before toe off and through swing phase in 60% BWS stepping, whereas during 20% BWS stepping, the VL activation decreased at mid swing phase. For participant 2, the RF activation remained constant through the transition from stance to swing in both 20% and 60% BWS stepping with activation higher during 60% BWS stepping. The VL activation increased prior to toe off and sharply decreased in swing phase, whereas in 20% BWS stepping VL activation decreased prior to toe off and remained decreased through swing phase. Overall, the activation of the RF and VL for both participants was greater during the transition from stance to swing phase during 60% BWS stepping compared to 20% BWS stepping, with the exception of the VL for participant 2. Changes between 20% and 60% BWS in the medial hamstring and distal muscles were minimal. Data can be viewed in the Supplementary Material.

## Discussion

During ES enabled BWST stepping in two participants with sensorimotor complete SCI, we demonstrate that descending intentional commands and afferent proprioceptive input result in varied modulation of the motor output identified through EMG and vGRF. Changes in stepping independence were described by the level of assistance perceived by the clinicians during BWST stepping, specifically during the transition from stance to swing. For both participants, optimal stepping performance requiring minimal trainer assistance was achieved when the participant was intentionally stepping (active stepping) and during trials when loaded with 80% of their body weight (20% BWS stepping). Conversely, when the participant was not intentionally stepping (passive stepping), or during trials when loaded with only 40% of their body weight (60% BWS stepping), the level of assistance increased during the transition from stance to swing. The increase in assistance was in response to an exaggerated lower extremity extension pattern demonstrated by a lack of lower extremity flexion necessary during the transition from stance to swing, likely caused by proximal extensor activation. In an effort to improve flexion of the lower extremity, substantial assistance from the clinician was needed at the posterior knee and the anterior ankle to facilitate swing phase.

### Influence of Descending Intentional Commands

For individuals with discomplete SCI, such as the two participants enrolled in this clinical trial, intentional participation can facilitate residual descending supraspinal input and engage remaining non-functional pathways to spinal circuitry below the injury when ES is applied (Dimitrijevic, 1988; Sherwood et al., 1992). Previous experiments in animal models with completely transected spinal cords have indicated restoration of stepping function and modulation of motor outputs despite the absence of supraspinal input (Lavrov et al., 2006; Courtine et al., 2009; Brand et al., 2012; Gad et al., 2013). However, SCI in humans is rarely a complete transection, and therefore, study participants likely have some remaining descending supraspinal input (Calvert et al., 2019b). Previous studies with non-invasive stimulation have indicated augmentation of motor outputs with voluntary input in individuals with motor incomplete SCI (Gerasimenko et al., 2015; Hofstoetter et al., 2015). While intentional participation seems logical during rehabilitation sessions, some continuous activities, such as BWST stepping, can become mundane and extremely taxing for both the study participant and the assisting clinicians. The effect of intentional motor commands using ES in motor complete SCI has not been described to this detail. Describing clinician assist during a specific phase of gait allows clinical interpretation of stepping performance while determining insufficient portions of the gait cycle. Previously, regaining intentional control of the lower extremities during ES-enabled stepping was specifically observed in the flexor muscles during intentional stepping (Angeli et al., 2014), however, these initial studies failed to demonstrate independent ES-enabled stepping, whereas recent publications have detailed independence (Angeli et al., 2018; Gill et al., 2018; Wagner et al., 2018). Functional gait must incorporate lower extremity flexion and extension patterns to be successfully independent. Here, we are describing modulation of the lower extremity extensor muscles responding to the descending intentional command to allow flexion to occur purposefully and with greater independence.

ES-enabled passive stepping describes the lack of intent, which resulted in tonic motor activity in the proximal lower extremity extensors, mostly impacting the transition from stance to swing phase. Additionally, passive stepping resulted in an increase in clinician perceived level of assistance likely due to the sustained knee extensor activity during co-contraction of the RF and VL, which impaired lower extremity flexion necessary to initiate swing and resulted in a prolonged stance phase with toe drag for both participants. Interestingly, participant 2 demonstrated an increase in load (vGRF) through the lower extremities during the stance phase during active stepping when compared to passive stepping. Thus, utilization of the upper extremities to assist with weight shifting and posture support did not result in an unloading response. Overall, while ES parameters remain unchanged between conditions, tonic extension patterns emerged during passive stepping supporting the need for intent to facilitate necessary modulation of the lower extremity muscles to improve independence during ES enabled BWST stepping.

### Influences of Afferent Proprioceptive Input

Afferent proprioceptive input during stepping has been shown to be critical to restore function in animal models of SCI (Lavrov et al., 2006). Additionally a meta-analysis of 54 studies of neurologically impaired and healthy human participants, investigating BWS adjustments and the effect on gait parameters including lower extremity motor activation, joint kinematics and kinetics, and vGRF, indicated afferent input had a strong influence on gait characteristics (Apte et al., 2018). The authors concluded that unloading (i.e., increasing BWS) of the lower extremities during BWST stepping reduced lower extremity motor activity; specifically, the mean activation of the lower extremity extensor muscles RF and SOL. Similar findings were reported by Harkema et al. (1997) with respect to motor output changes during alterations in loading as well as muscle-tendon stretches in the SCI population, which resulted in decreased motor activity of distal leg muscles, specifically the MG and the SOL, when unloading increased.

However, we report the opposite phenomenon, a decrease in motor output compared to an increase during supra-motor threshold ES-enabled BWST stepping with motor complete SCI individuals under conditions identified as ideal for promoting greater independence from clinician assist. Participant 1 exhibited increased motor activation of the RF muscles during stance at 60% BWS stepping compared to 20% BWS stepping and greater activation of the RF and VL during swing at 60% BWS stepping compared to 20% BWS stepping. Participant 2 experienced the same phenomenon in both the RF and VL, demonstrating greater motor activity during stance and swing under 60% BWS stepping conditions. Even when motor activity decreased during 20% BWS stepping, the level of assistance perceived by the clinician did not increase, implying the motor activation was sufficient to maintain knee extension for the load applied. Stepping with 20% BWS resulted in the greatest level of independence for both participants. Whereas, 60% BWS stepping resulted in greater clinician assistance during the transition from stance to swing transition phase due to the sustained activity of proximal knee extensor muscles. Demonstrated through vGRF, lower extremity loading was greater during 20% BWS stepping compared to 60% BWS stepping which is to be expected; however, during the transition from stance to swing, participant 2 had a considerably longer stance phase even with less loading through the limb. This exaggerated stance phase required an extreme increase in clinician assist, likely due to the amount of leg extension prohibiting flexion.

During ES-enabled BWST training, emphasis was placed on decreasing BWS while encouraging participants to use of their upper extremities, placed on rigid parallel bars, in order to maintain appropriate posture throughout their trunk and pelvis during stepping. More specifically, upper extremity use of the parallel bars allowed manipulation of body positioning and weight shifting throughout the step cycle, such as facilitating hip and trunk extension in order to initiate stance and/or swing phases. We recognize that this compensatory strategy has the ability to impact lower extremity loading during stance, however, these changes in vGRF were not observed in the present study. Interestingly, we observed that use of the upper extremities may have positively impacted stepping independence by facilitating appropriate trunk and pelvic positioning, specifically during intent conditions. We surmise this observation may be due to changes in lower extremity and trunk afferent signaling such as kinematic muscle stretching at the hips. Likewise, in the absence of upper extremity-induced manipulation, specifically during the transition from stance to swing phase, the hip flexor muscles may have not received sufficient afferent input of a stretch, in turn, resulting in inadequate afferent signaling necessary to cue hip flexor activity.

### Potential Strategies to Enhance Performance During ES-Enabled BWST Stepping

Based on the findings presented in this paper, we offer strategies to facilitate increased independence of performance for individuals with SCI during ES-enabled BWST stepping. ES-enabled BWST stepping performed in an environment that emphasizes intentional participant involvement, using real-time visual and verbal feedback regarding performance of stepping, enables greater independence. During training we recommend maximizing weight-bearing through the lower extremities by decreasing BWS as much as possible to promote convergence of the afferent proprioceptive input and descending intentional commands. Achieving optimal performance may require participants to compensate in other areas (e.g., use of arms on parallel bars) to allow afferent input from lower extremity loading and ES to converge facilitating adequate motor output necessary for stepping. Prior evidence generated in animal models of SCI (Gad et al., 2013; Wenger et al., 2014, 2016; Capogrosso et al., 2016; Islam et al., 2019; Chia et al., 2020), as well as in humans by our research team and others, indicates the importance that individualized ES parameters in order to enable intra-limb rhythmic motor activity of the lower extremities, and in turn restore stepping (Harkema et al., 2011b; Minassian et al., 2013; Hofstoetter et al., 2015; Grahn et al., 2017; Angeli et al., 2018; Gill et al., 2018; Wagner et al., 2018; Calvert et al., 2019a).

### Limitations/Future Directions

Given the heterogeneity of severity of SCI, and limited sample size, we recognize that our results, along with previously reported literature, may not be generalizable to all individuals with SCI. A high degree of variability was seen between our two participants, signifying the necessity for further assessment of afferent proprioceptive input and descending intentional command changes across many individuals with various severities of SCI while using ES. Quantifying the clinician's level of assistance was subjective; however, utilizing quantitative measures to determine the level of assistance would add value to the interpretation of performance. Additional biomechanical assessments of BWST stepping would strengthen the understanding of motor coordination while a longitudinal comparison of EMG and vGRF may describe a training effect.

In conclusion, during ES-enabled BWST stepping participant intent and BWS modification can impact motor output, vGRF, as well as performance. ES-enabled motor activation facilitating independence of stepping requires input above and below the level of injury to facilitate modulation of specific muscle groups to improve performance, specifically during the transition from stance to swing during gait.

## Data Availability Statement

The raw data supporting the conclusions of this article will be made available by the authors, without undue reservation.

## Ethics Statement

The studies involving human participants were reviewed and approved by Mayo Clinic IRB. The patients/participants provided their written informed consent to participate in this study. Written informed consent was obtained from the individual(s) for the publication of any potentially identifiable images or data included in this article.

## Author Contributions

MG, PG, DS, and KZ initiated the project. MG, PG, ML, and AT designed the experiments with contributions from all authors. LB, KG, MG, PG, IL, ML, and DV performed clinical assessments. LB, JC, MG, PG, ML, and DV designed and performed rehabilitation. LB, JC, MG, PG, ML, IL, and DV contributed to stimulation setting refinement. LB, JC, KF, MG, PG, RH, ML, CL, AT, DV, and KZ contributed to data collection, analysis, and interpretation. KF, MG, PG, RH, and ML drafted the manuscript with subsequent contribution from all authors. KZ supervised all aspects of the work. All authors contributed to the article and approved the submitted version.

## Conflict of Interest

The authors declare that the research was conducted in the absence of any commercial or financial relationships that could be construed as a potential conflict of interest.
