# Supplementary material for: Alterations of Spinal Epidural Stimulation-Enabled Stepping by Descending Intentional Motor Commands and Proprioceptive Inputs in Humans With Spinal Cord Injury
Source: Front Syst Neurosci. 2021 Jan 28;14:590231. doi: 10.3389/fnsys.2020.590231 (PMC7875885; doi:10.3389/fnsys.2020.590231)
Supplement: Supplementary file 1 [file Table_1.pdf]

SUPPLEMENTAL FIGURE DESCRIPTIONS

Supplemental Figure 1. Participant 1 left and right side individual muscle electromyogram results during passive (gray) and active (blue) BWS stepping. Continuous data is represented by the mean ± SD of the linear envelope throughout the gait cycle. The dashed line represents the mean time point of transition from stance to swing phase. Bar graphs represent the difference in EMG amplitude between passive and active stepping at 10% intervals of the gait cycle. All data is separated by side and muscle group.

Supplemental Figure 2. Participant 1 left and right side mean RMS results separated by stance and swing phase during passive (gray) and active (blue) stepping. Error bars represent one standard deviation.

Supplemental Figure 3. Participant 1 left and right side individual muscle electromyogram results during 60% (gray) and 20% (blue) BWS stepping. Continuous data is represented by the mean ± SD of the linear envelope throughout the gait cycle. The dashed line represents the mean time point of transition from stance to swing phase. Bar graphs represent the difference in EMG amplitude between 60% and 20% BWS stepping at 10% intervals of the gait cycle. All data is separated by side and muscle group.

Supplemental Figure 4. Participant 1 left and right side mean RMS results separated by stance and swing phase during 60% (gray) and 20% (blue) BWS stepping. Error bars represent one standard deviation.

Supplemental Figure 5. Participant 2 left and right side individual muscle electromyogram results during passive (gray) and active (blue) BWS stepping. Continuous data is represented by the mean ± SD of the linear envelope throughout the gait cycle. The dashed line represents the mean time point of transition from stance to swing phase. Bar graphs represent the difference in EMG amplitude between passive and active stepping at 10% intervals of the gait cycle. All data is separated by side and muscle group.

Supplemental Figure 6. Participant 2 left and right side mean RMS results separated by stance and swing phase during passive (gray) and active (blue) stepping. Error bars represent one standard deviation.

Supplemental Figure 7. Participant 2 left and right side individual muscle electromyogram results during 60% (gray) and 20% (blue) BWS stepping. Continuous data is represented by the mean ± SD of the linear envelope throughout the gait cycle. The dashed line represents the mean time point of transition from stance to swing phase. Bar graphs represent the difference in EMG amplitude between 60% and 20% BWS stepping at 10% intervals of the gait cycle. All data is separated by side and muscle group.

Supplemental Figure 8. Participant 2 left and right side mean RMS results separated by stance and swing phase during 60% (gray) and 20% (blue) BWS stepping. Error bars represent one standard deviation.
