# Supplementary figures and images for: Alterations of Spinal Epidural Stimulation-Enabled Stepping by Descending Intentional Motor Commands and Proprioceptive Inputs in Humans With Spinal Cord Injury
Source: Front Syst Neurosci. 2021 Jan 28;14:590231. doi: 10.3389/fnsys.2020.590231 (PMC7875885; doi:10.3389/fnsys.2020.590231)

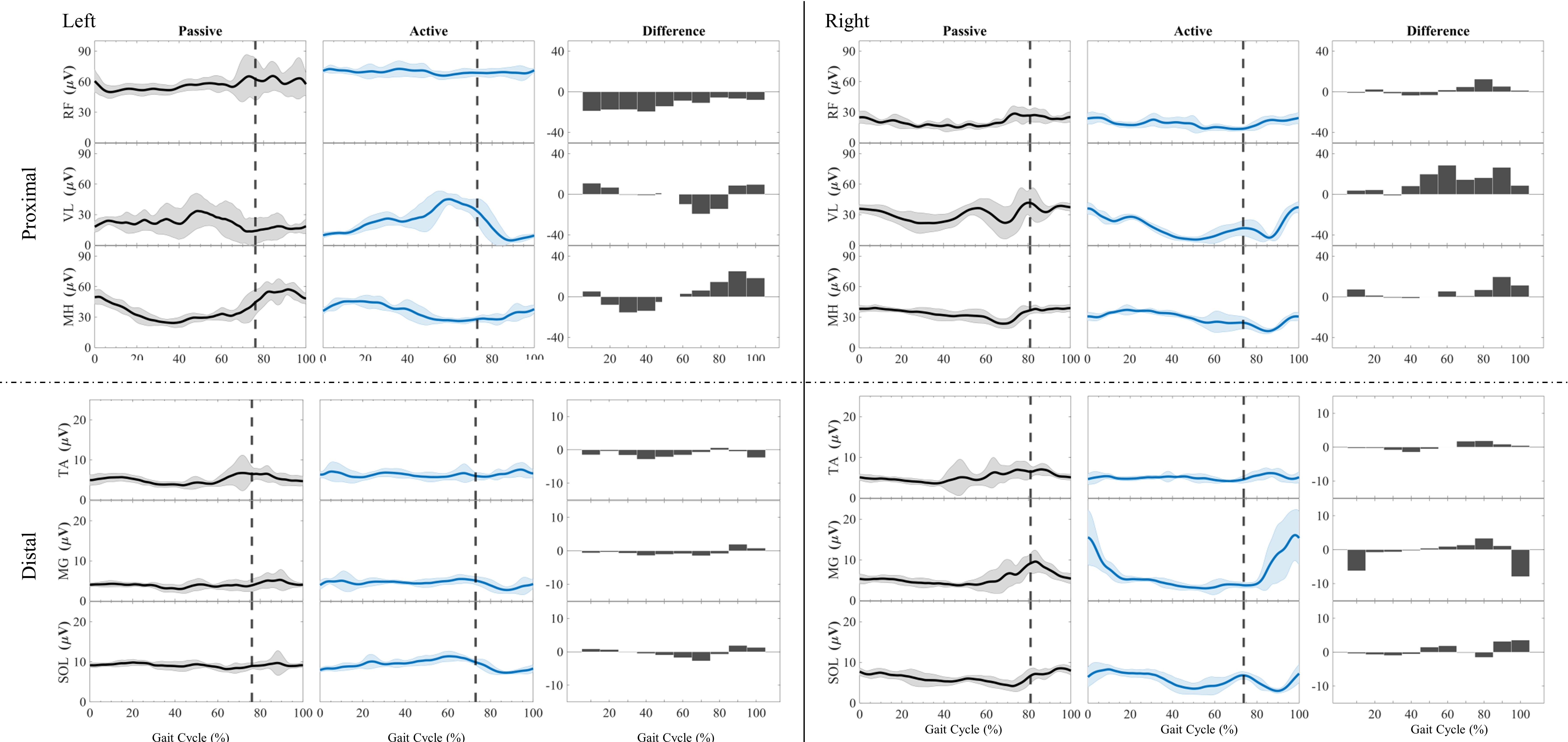

Supplement: Supplementary file 2 [file Image_1.JPEG]

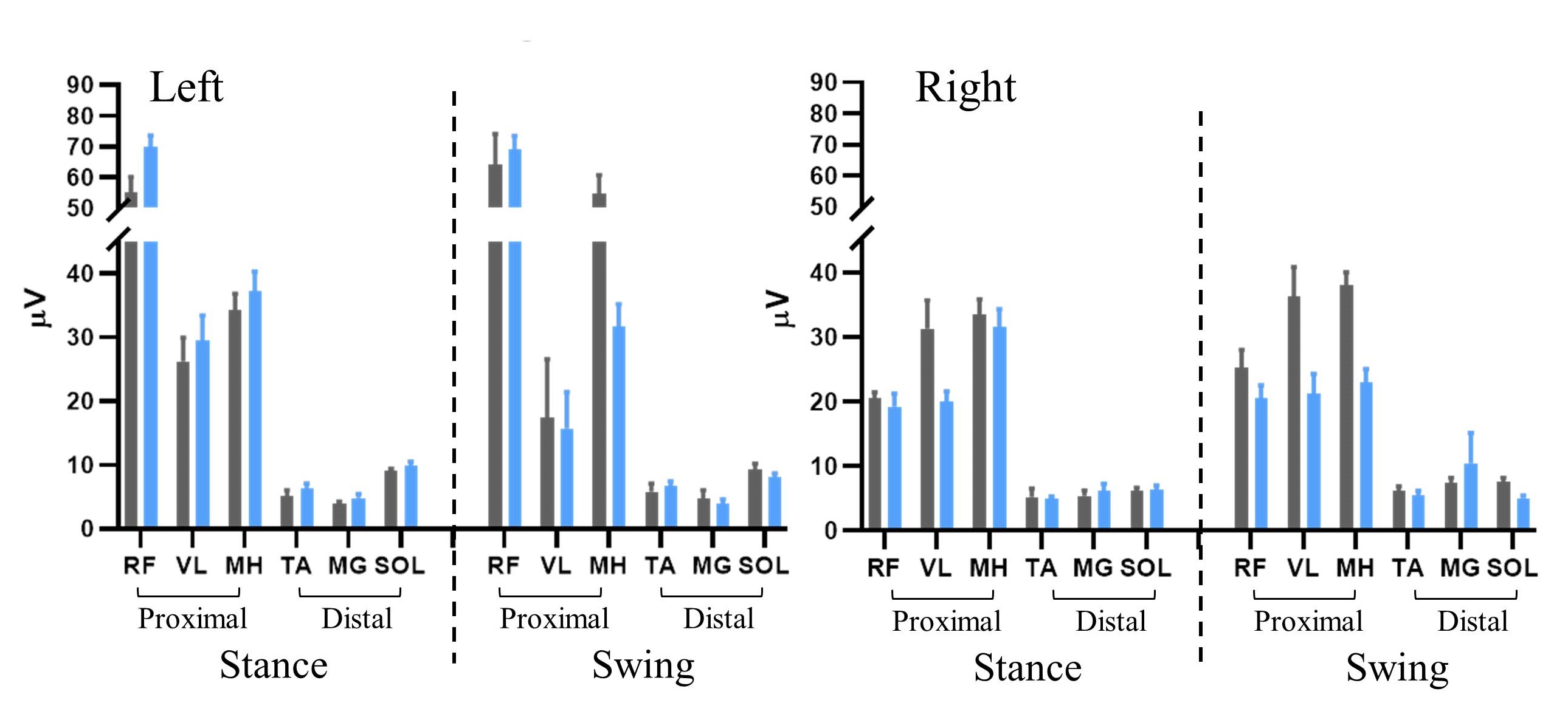

Supplement: Supplementary file 3 [file Image_2.JPEG]

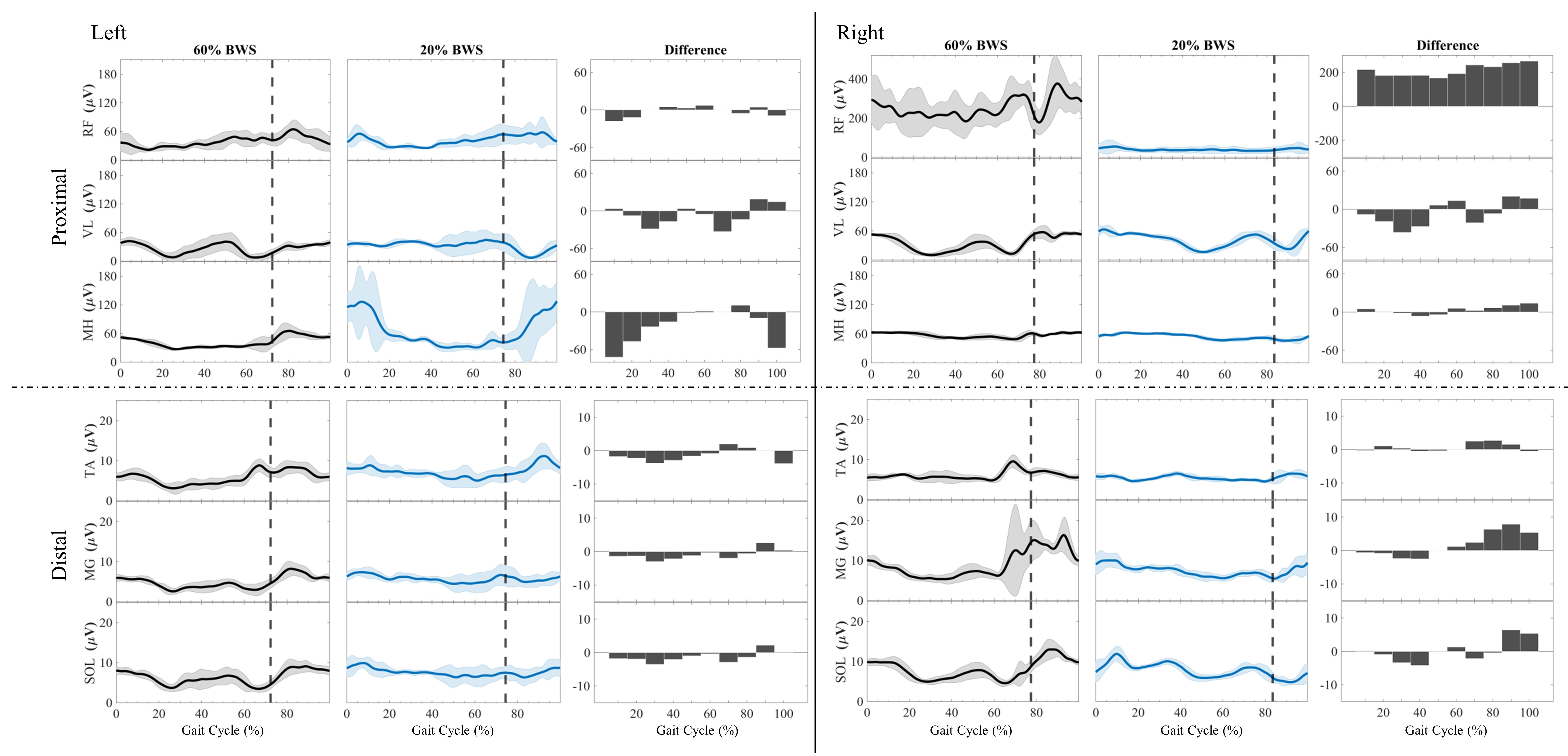

Supplement: Supplementary file 4 [file Image_3.JPEG]

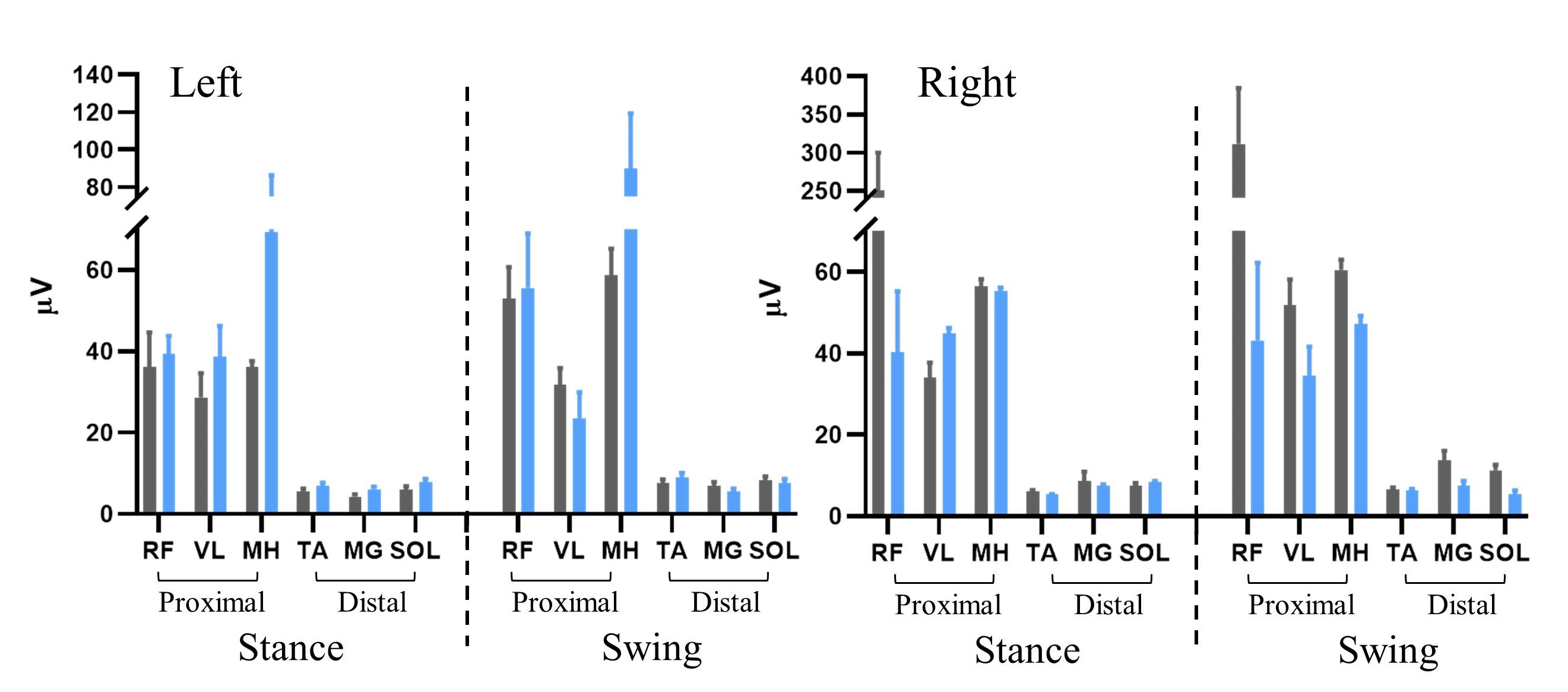

Supplement: Supplementary file 5 [file Image_4.JPEG]

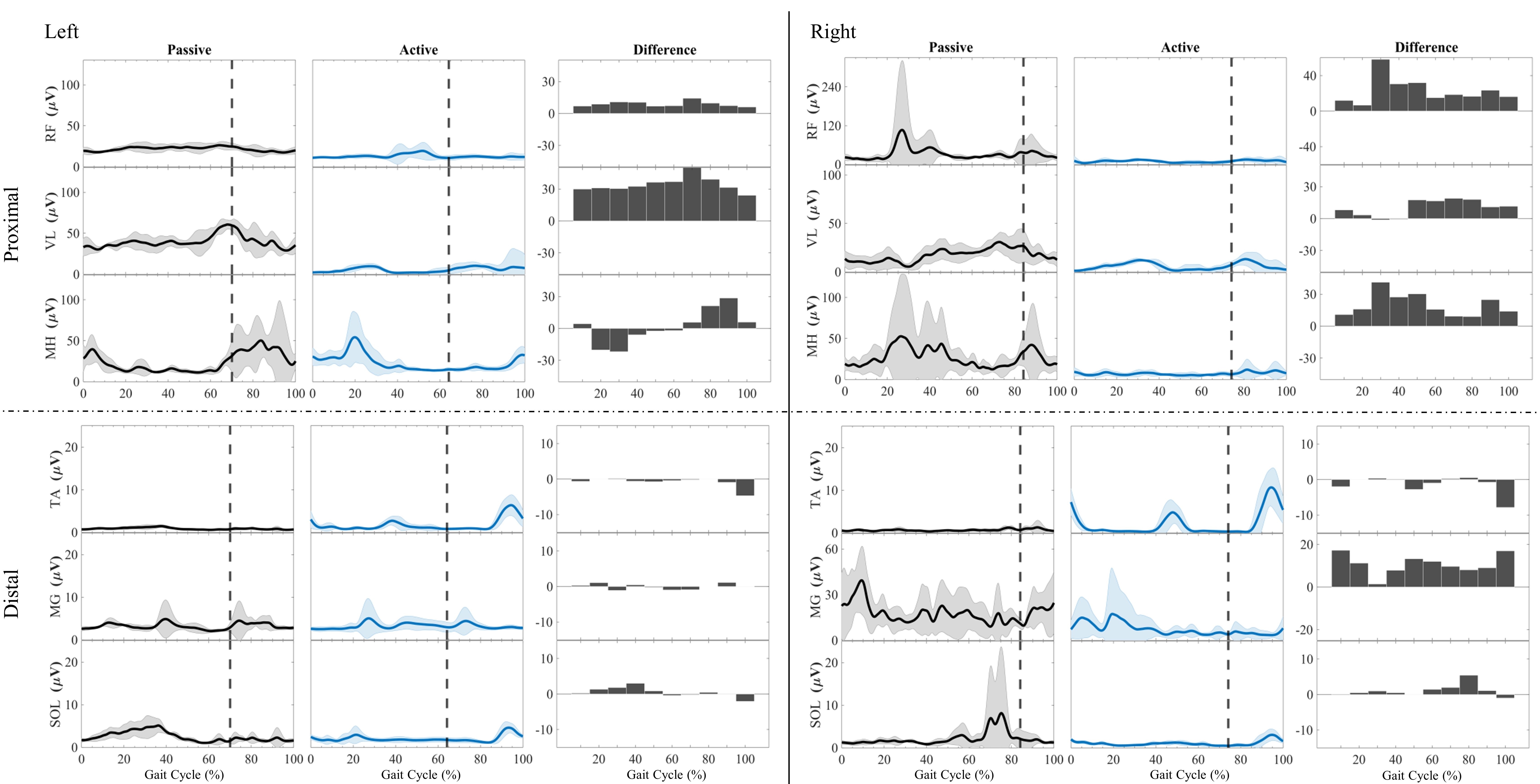

Supplement: Supplementary file 6 [file Image_5.JPEG]

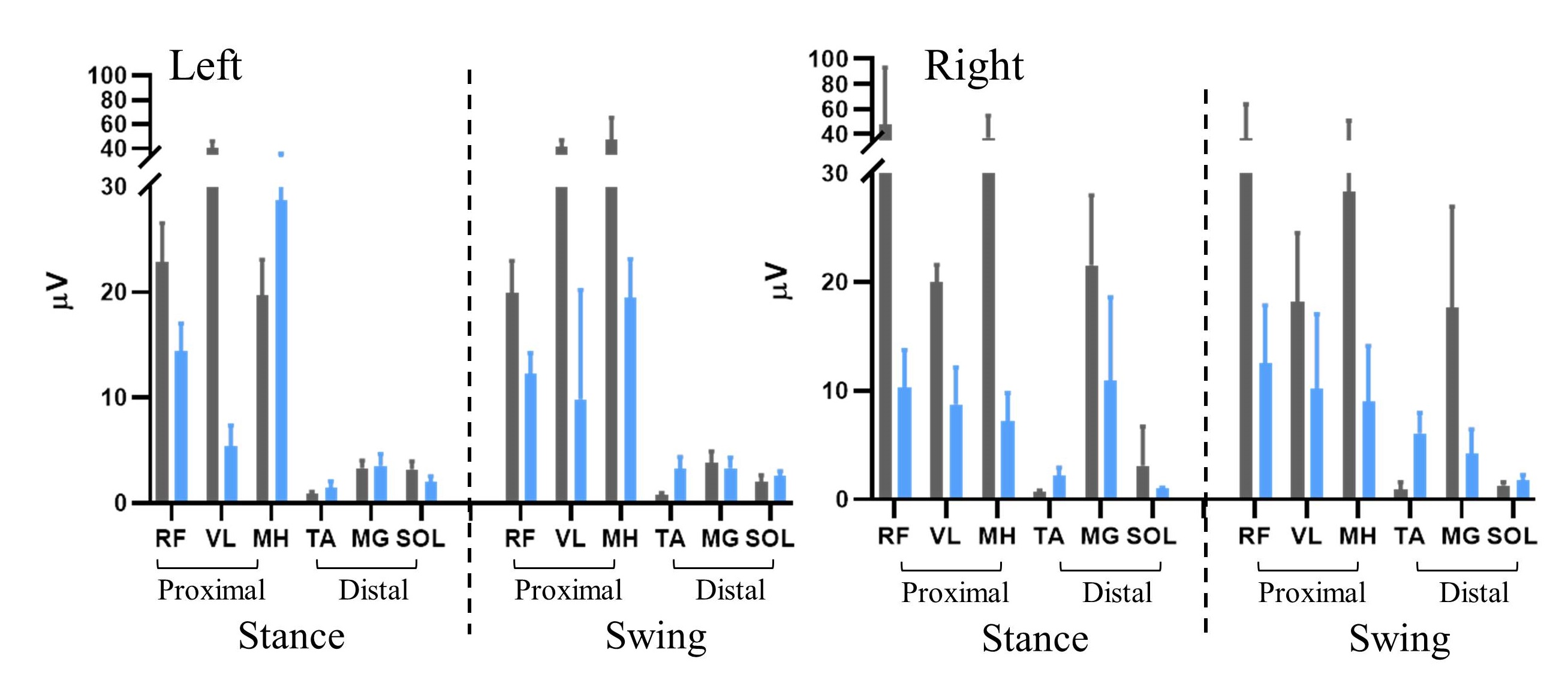

Supplement: Supplementary file 7 [file Image_6.JPEG]

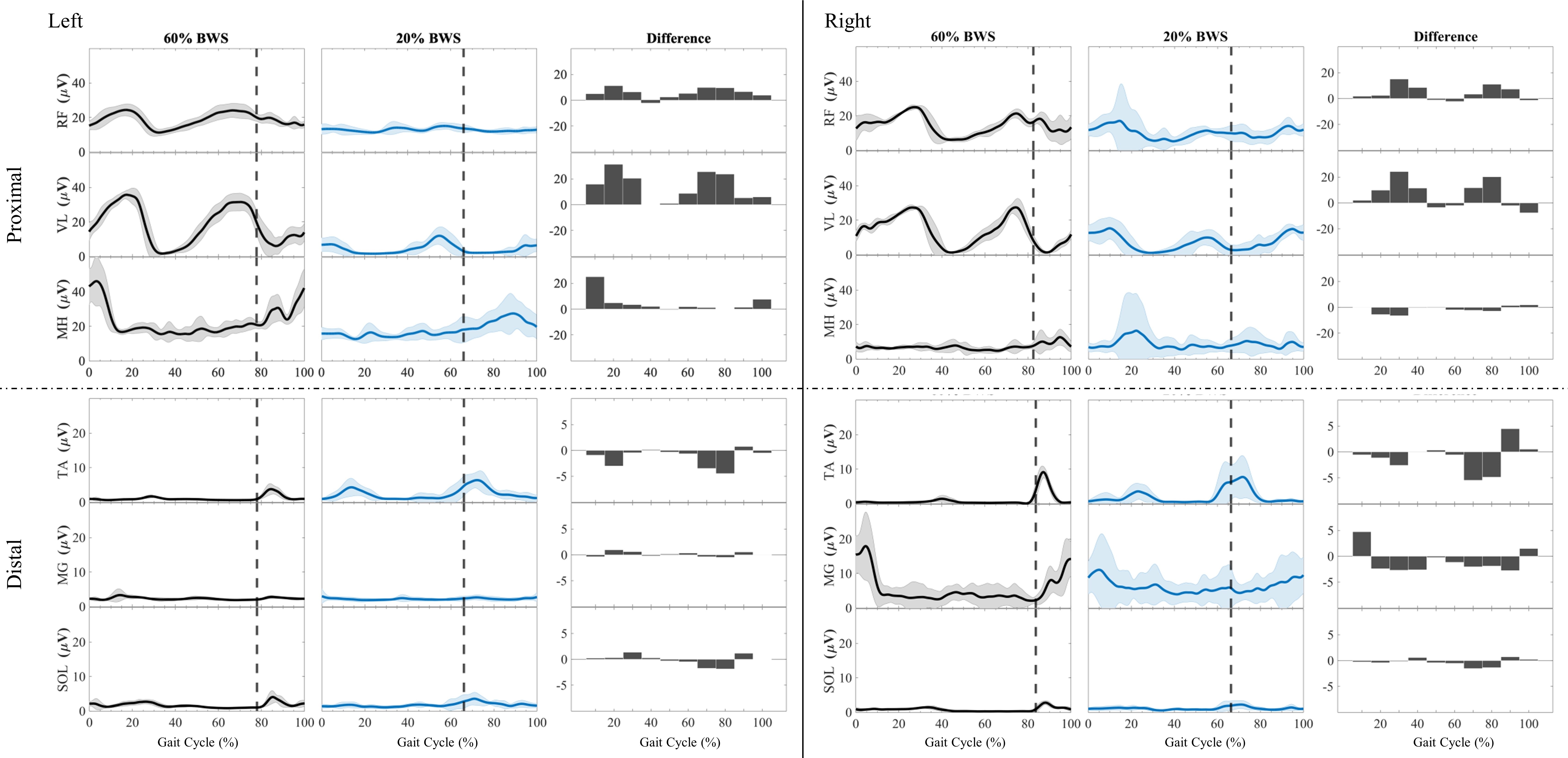

Supplement: Supplementary file 8 [file Image_7.JPEG]

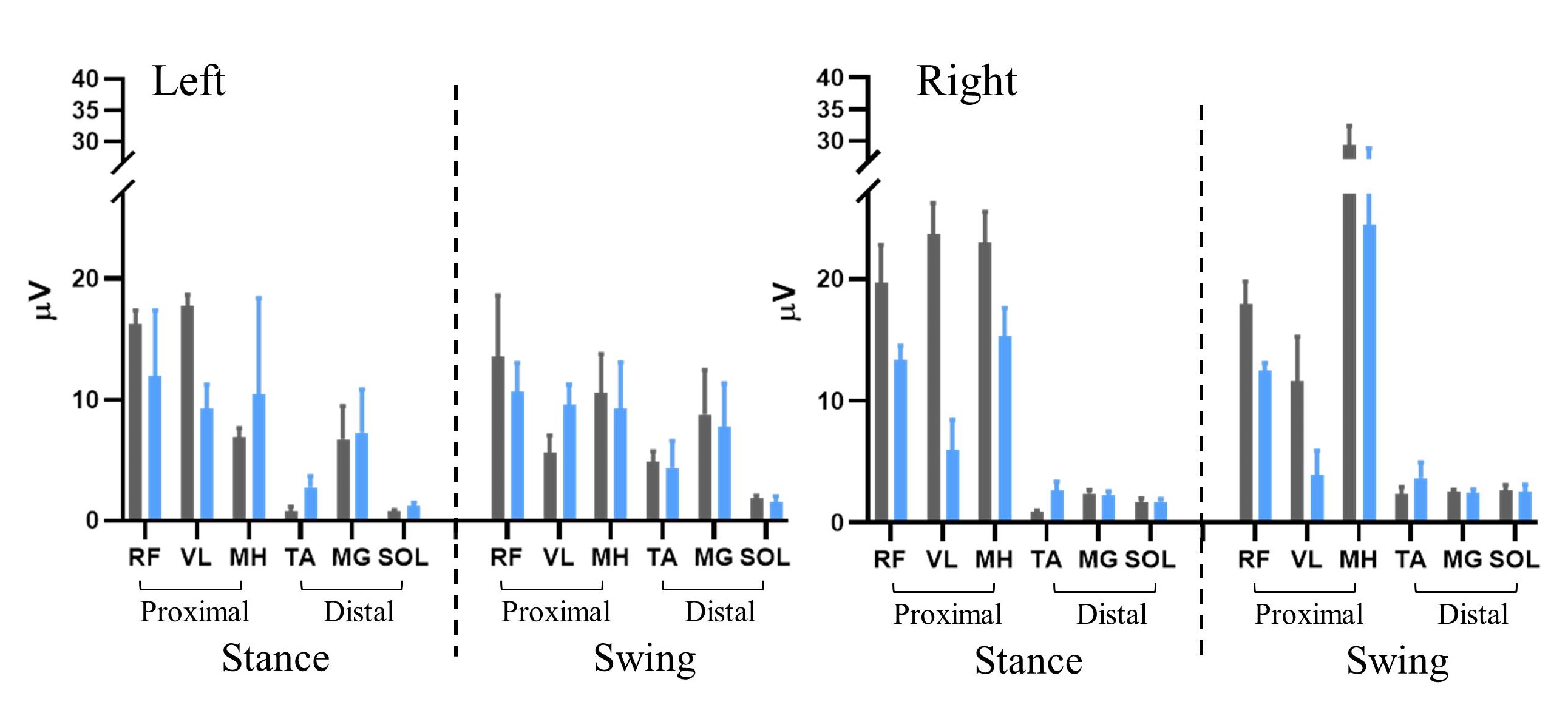

Supplement: Supplementary file 9 [file Image_8.JPEG]
